# A Scoping Review of Literacy Interventions Using Signed Languages for School-Age Deaf Students

**DOI:** 10.3390/bs15081104

**Published:** 2025-08-14

**Authors:** Hannah M. Dostal, Jessica A. Scott, Marissa D. Chappell, Christopher Black

**Affiliations:** 1 Department of Curriculum and Instruction, Neag School of Education, University of Connecticut, Storrs, CT 06269, USA; marissa.chappell@uconn.edu; 2 Department of Learning Sciences, College of Education & Human Development, Georgia State University, Atlanta, GA 30302, USA; jscott96@gsu.edu (J.A.S.); cblack4@student.gsu.edu (C.B.)

**Keywords:** sign language, literacy, reading, writing

## Abstract

This scoping review systematically analyzes the nature and implications of existing research on literacy and literacy interventions that use a signed language among deaf students from preschool through college. We identified the findings associated with the use of sign languages on literacy outcomes for deaf students, and we analyzed the scope of the identified studies to uncover potential gaps in the research base. Fifteen empirical studies published between 2014 and 2025 met the inclusion criteria, featuring participants across a range of educational levels. Methodologies varied, with qualitative and group comparison designs most common. Studies addressed key literacy components (i.e., phonological awareness, fluency, vocabulary, comprehension, and composition) and findings indicate that integrating sign language into literacy instruction enhances language access and supports literacy learning. The results highlight the importance of responsive, multimodal instruction and point to the need for further research to fill identified gaps.

## 1. Literature Review

### 1.1. Sign Language Research

Research on signed languages has a relatively recent but rapidly evolving history within the broader fields of linguistics, psychology, education, and cognitive sciences (Sandler & Lillo-Martin, 2006). Although sign languages have been used in deaf communities for centuries, formal research into their structures, acquisition, and use did not gain momentum until the mid-20th century (Stokoe, 1960). Early recognition of sign languages as natural languages, such as through foundational linguistic analyses of American Sign Language (ASL) in the 1960s, contributed to a broader shift in perception, positioning signed languages as complex and fully developed linguistic systems (Stokoe, 1960; Klima & Bellugi, 1979). This shift occurred in parallel across several national contexts (Sutton-Spence & Woll, 1999) as researchers documented and described the structure and use of diverse sign languages around the world (Pfau et al., 2012).

As research on sign languages has grown, so too has recognition of the linguistic diversity within signing communities worldwide. Current estimates suggest that there are between 150 and 300 distinct signed languages used globally, depending on how languages are categorized and documented. Ethnologue (www.ethnologue.com), a leading linguistic database, lists approximately 144 to 150 signed languages that the meet criteria for International Organization for Standardization recognition (i.e., ISO 639-3), reflecting only well-documented systems (Eberhard et al., 2025). However, broader linguistic and sociocultural estimates from organizations such as the World Federation of the Deaf (WFD) and the United Nations place the number closer to 200–300, as they include emerging and less formally documented languages, such as local sign languages or national varieties not yet cataloged by international standards (World Federation of the Deaf, 2018; United Nations, 2023). Cross-linguistic academic projects like the SIGN-HUB Atlas (thesignhub.eu) further suggest that the number of distinct sign languages continues to grow, especially as research expands to underserved regions and historically excluded communities (Quer & Cecchetto, 2020). This global linguistic diversity is critical for understanding both the scope of sign language research and the varied contexts in which individuals acquire and use sign languages.

Over time, research on sign languages expanded beyond structural linguistics to include acquisition, multilingualism, neurolinguistics, sociolinguistics, and education. Much of the early research focused on adult native signers, often those who had deaf parents (Newport, 1990; Petitto, 1987). This population became the primary reference group for many theoretical and empirical models of sign language proficiency, acquisition and processing. However, as the field matured, it became increasingly clear that native signers represent a small portion within the larger deaf signing population. Estimates indicate that fewer than 10% of deaf children are born to deaf parents (Mitchell & Karchmer, 2004), meaning that the majority of sign language users acquire sign later in life and often under variable conditions of access and exposure (Emmorey, 2002; Mayberry & Eichen, 1991). This demographic reality has prompted critical shifts in research agendas to account for the broader diversity in language backgrounds, age of acquisition, and educational experiences among deaf and hard-of-hearing signers. As a result, more recent work has begun to consider how late exposure to sign language (Hall, 2017; Hall et al., 2017) and varied access to signing environments (Holcomb et al., 2024; Humphries et al., 2014) shape both linguistic development and performance outcomes.

There has also been increasing attention to cross-linguistic studies involving different sign languages, the development of assessments tailored to diverse signing populations (Hoffmeister, 2000), and the sociopolitical contexts that impact sign language use, recognition, and transmission (Lane et al., 1996; Lucas, 1995). Together, these trends underscore the importance of situating research about signed languages in the demographic and lived experiences of the communities that use these languages. These developments also provide essential context for the present scoping review, which seeks to examine how literacy research reflects the linguistic realities of signing deaf learners.

### 1.2. Literacy and Deaf Children

Though literacy development and outcomes among deaf students is perhaps the most often studied area in deaf education research, our knowledge in this realm is still limited. For many years, research in the U.S. was preoccupied with the status of ASL as a language, or with making comparisons between the literacy outcomes of deaf learners and a comparison group of hearing learners (see Holcomb et al., 2024, for a discussion of why such comparisons can be harmful rather than useful). Outside of these topics, ultimately there are few articles that engage with the development of literacy skills for deaf students (Gabriel et al., 2025). Below, we explore two major topics relevant for this review: The first is the role of cognition within the literacy of deaf learners, and the second is related to literacy strategies.

#### 1.2.1. Literacy and Cognition

For all learners, there is a reciprocal relationship that exists between literacy and cognition—improvements in one area tend to be accompanied by improvements in the other. This holds true for students with a range of disabilities, most commonly attention deficit hyperactivity disorder (Felton & Wood, 1989; McDougal et al., 2021) and intellectual disability (Nilsson et al., 2025). Cognitive areas that appear to be most closely associated with literacy skills include working memory, rapid naming, and short-term memory (Felton & Wood, 1989; McDougal et al., 2021; Swanson et al., 2012).

Unfortunately, there has been little research into the specific dimensions of cognition of deaf learners and their relationship with literacy. Martin (1993) suggested that deaf learners have as much capacity for cognitive development as hearing children, an assertion with which we certainly agree. However, it is essential to note that many deaf learners are not given an equal opportunity to develop these cognitive abilities due to the rate at which deaf children experience language deprivation (Hall et al., 2017). This may explain the differences noted in one study comparing various cognitive skills of deaf and hearing learners in areas such as visuo-spatial processing, memory, and executive function (Marschark & Knoors, 2012). Though, following Holcomb et al.’s (2024) critiques of comparison studies, we note the inherent problems in comparing potentially multilingual deaf students to likely monolingual hearing students. We also concur with Marschark and Knoors’ conclusion that differences in how information may be processed between deaf and hearing learners is information that is valuable for teachers working with these students.

One study had as an explicit aim the exploration of cognitive and literacy tasks among deaf learners (Kyle & Harris, 2011). However, the authors appeared to only include one non-language specific cognitive task (short-term memory), and several of the remaining tasks are ones that we would consider linguistic. Not only that, but they are linguistic tasks that are specifically oriented towards spoken language in particular (e.g., phonological awareness, speechreading, letter sound knowledge). Unsurprisingly, deaf students performed more poorly in most of these sound-based tasks when compared to hearing students. The only cognitive task included, short term memory, was only correlated with knowledge of letter names for the deaf participants. Overall, we emphasize that few studies explore the cognitive development of deaf learners, and even fewer do so in a way that is not specifically tied to a spoken language. As a result, our knowledge of cognition and literacy as it specifically relates to a visual language such as ASL is limited. This is the area we explore in this scoping review.

#### 1.2.2. Literacy, Sign Language, and Print

One important metric for examining the impact of instruction in various literacy skill areas on overall literacy growth is through intervention-based research (see Dostal & Lederberg, 2020 for a discussion). Such studies test the efficacy of teaching participants a particular skill via a particular method, and then measure growth in this area. Research on literacy interventions has spanned populations such as young children (Justice et al., 2003; Wasik et al., 2006), students with disabilities (Allor et al., 2020; Koppenhaver et al., 2007; Machalicek et al., 2009), and minority students/students of color (Beach et al., 2018; Walker & Hutchison, 2020). These types of studies are essential because they provide tangible evidence for how to support students as they develop this complex array of skills.

The concept of literacy often has varying definitions across fields of study, political climates, and cultural or historical contexts (Keefe & Copeland, 2011). This reflects differing priorities regarding what it means to be “literate” in a given society, according to their respective values (Gee, 2015; Street, 1984). Additionally, the scope of the definition of literacy can range from individualized and skill-based approaches (Copeland & Keefe, 2007; Katims, 1994; Kliewer et al., 2006; Mirenda, 2003; UNESCO, 2017) to broader conceptual and social frameworks (Barton & Hamilton, 1998; Perry, 2012). Some definitions of literacy even expand to specific content areas, and delineate both broad and narrow skills required for literacy in that content area (National Research Council, 1996). In today’s modern technological climate, the definition of literacy in the academic sense often converges the multiple meanings of the term itself. Typically expanding to overlap with terms like ‘digital literacy’, ‘information literacy’ and ‘multiliteracies’ that are necessary to be an engaged citizen in today’s context (Pilgrim & Martinez, 2013). All of these terms describe skills needed for information exchange and learning in a modern era, including but not prescriptively centering or privileging traditional modalities such as reading and writing (Karkar Esperat, 2024). Use of an asset-based lens and multiliteracies pedagogy has been supported to foster student agency, language and literacy development, affirmation of cultural identity, critical literacy, and student engagement and collaboration for multilingual English Language Learners (Rajendram, 2016).

Additionally, beliefs about literacy influence both classroom practices (Ungureanu, 2024) and research methodologies (Keefe & Copeland, 2011). It is beyond the scope of the present review to establish an epistemologically stable definition of literacy; rather, we aim to underscore the variability of the term across contexts. Many of the studies included in this review operate under differing conceptualizations of literacy, each of which forms the foundation for their research design and interpretation of outcomes. These underlying definitions and informative beliefs inevitably shape the research questions posed, the interventions selected, and the metrics used to assess effectiveness. As such, these embedded assumptions introduce biases that complicate direct comparisons across studies. This should be carefully considered when evaluating the results of the studies introduced in this review.

Intertwined with complex definitions of literacy include broadened, multi-faceted and context-dependent definitions of “text” that are reliant on their governing pedagogical foundations. Hobbs (2016) effectively articulates that as the term “literacy” began evolving to include “practices of writing, speaking, and listening” the term “text” has evolved to be understood as “symbolic representations in any of a number of forms, including spoken and printed language, still and moving images, sound and multimedia” (pg. 1). Under this definition, a video recorded publication of a work produced in American Sign Language (ASL) would be considered a text, and the ability to view, interpret, engage with, and make meaning from that text would be considered an appropriately applied form of literacy in ASL.

Literacy in ASL has a similarly complex history in the literature (Zernovoj, 2015). This includes varying references to “ASL literacy” as merely fluent and competent language use (Byrne, 2013) to the rich histories and literature passed down via indigenous languages, such as ASL, despite their lack of written documentation (Christie & Wilkins, 1997). This revisits the concept of literacy as a social practice that requires fluency in a given language to be complete, while also emphasizing the development of the ability to critically engage with permanently documented content, or “text”, in a heritage language. Byrne (2013) sought to categorize ASL texts according to their own respective genres which further supports the conceptualization of literacy in ASL as competence in linguistic and social practices within deaf communities, and ability to critically engage with the content produced by that community.

#### 1.2.3. Cross-Linguistic Transfer and Justification for Signed Interventions to Support Print Literacy

It is with this foundation that educational resources like the ASL Content Standards were developed, to support skill development of students in their ability to view literature and informational texts that were video recorded in ASL as well as their skills in the creation of a published work in ASL that are separate and distinct from ASL or English language fluency standards necessary for educational achievement (Clerc Center & California Department of Education, 2020). Allowing students to engage in both viewing and composition in their native language supports underlying linguistic skills required for skilled print reading (Bernhardt, 2010; Mayberry & Chamberlain, 2008). A strong first language (L1) supports second language (L2) learning by serving as a foundation for mapping English print forms, providing essential background knowledge for reading comprehension, enabling effective instruction, and helping learners grasp metalinguistic concepts like grammar and word meaning (Hoffmeister & Caldwell-Harris, 2014; Caldwell-Harris & Hoffmeister, 2022). Deaf ASL-English bilinguals demonstrate automatic and efficient cross-linguistic activation of ASL during English word recognition. For example, lexical decision tasks show faster and more accurate responses when English words are phonologically or semantically related to their ASL translations in both adults (Morford et al., 2011) and children (Villwock et al., 2021). Similar findings have been observed across different methodologies, including eye-tracking studies that reveal predictive gaze behavior indicative of sign-based semantic activation (Saunders et al., 2024), and event-related potential (ERP) studies demonstrating early neural activation of ASL equivalents during English lexical processing (Bosworth et al., 2021).

These findings challenge the notion that ASL is unrelated to English literacy and instead position ASL as a foundational linguistic resource that can scaffold and enhance the acquisition of English skills acquired through print in deaf bilingual children. Several empirical studies have demonstrated that ASL proficiency and ASL-based instructional interventions can significantly support the development of English reading skills in school-age deaf children. By leveraging a signed language for the deaf signing child, sign-language-based interventions can provide linguistically and culturally accessible ways to support cognitive and linguistic processes that are transferable to English print literacy. For example, narrative structure (Hoffmeister, 2000; Kuntze, 2004), vocabulary depth (Enns et al., 2016; Hrastinski & Wilbur, 2016), metalinguistic awareness (Hoffmeister & Caldwell-Harris, 2014; Allen et al., 2014), and cross-modal language mapping (Chamberlain & Mayberry, 2000; Morford et al., 2011; Bosworth et al., 2021).

Despite this promising theoretical basis, there is considerable variability in how interventions that use signed languages are designed and implemented, as well as in the outcomes they report. Our scoping review was therefore motivated by the need to systematically examine this heterogeneity by investigating the range of intervention approaches and the literacy-related results they have yielded in light of the cross-linguistic relationship between ASL and English.

Despite increasing attention to deaf learners’ linguistic and cognitive experiences, research on literacy development that meaningfully incorporates sign languages lacks clear boundaries. Given this, a scoping review can bring together this work by systematically mapping the landscape of existing research, assess its methodological and conceptual orientations, and identify areas in need of further exploration. This review aims to highlight patterns and gaps in the literature in order to inform future research and educational practice that better reflects the realities of signing deaf learners.

### 1.3. Positionality

Our research team approached this scoping review with a shared commitment to valuing deaf individuals and recognizing the diversity within deaf communities. We acknowledge that our identities and professional experiences influence how we engage with the literature. Our team includes researchers who identify as hard of hearing and hearing; we bring diverse experiences in bilingual and multilingual deaf education, clinical practice, ASL interpreting, and family engagement across multiple languages (i.e., ASL, English, Spanish). While our backgrounds vary, we share a dedication to critically examining the role of sign languages in literacy development and to elevating research that reflects the linguistic and cultural experiences of deaf learners.

As a team, we sought to engage with the literature to answer the following research question: What is the nature of published research exploring the relationship between sign languages and literacy for deaf learners? Specifically, we were interested in studies that included an intervention or strategy to impact the development of literacy skills of participants.

## 2. Methods

We conducted a scoping review to analyze the nature and implications of existing research on literacy among signing deaf students from preschool through college. Our goal was twofold: first, to identify the findings and implications associated with the use of sign languages on literacy outcomes for deaf students; and second, to analyze the scope of the identified studies to uncover potential gaps in the research base. A scoping review was appropriate given the limited and heterogeneous nature of the evidence, allowing us to map existing research and clarify the state of the field, objectives that align with the intended purpose of scoping reviews (Munn et al., 2018). In contrast, a systematic review would have been premature, as it requires a more robust and consistent body of evidence to support synthesis or educational and clinical decision-making.

### 2.1. Protocol

The protocol for identifying and including articles in this study was developed using the Preferred Reporting Items for Systematic Reviews and Meta-Analyses (PRISMA) framework. It was refined through two iterative phases. The first two authors conducted searches and applied the protocol to a sample of three articles. Based on their findings, they revised the search terms and protocol parameters, then shared the parameters with the third and fourth authors, who independently applied the protocol and provided feedback. The final protocol, detailed below, reflects the consensus of all researchers.

To meet the inclusion criteria, articles needed to demonstrate a clear focus on any aspect of literacy (i.e., any domain of reading or writing), include deaf participants in preschool through postsecondary education, and use a signed language. Articles that were not focused on the use of a signed language specifically during the intervention process were not included here; likewise, those that examined the relationship between a signed language and literacy but did not intervene on these skills were also not included. Peer-reviewed academic journal articles were included if they were aligned with the above criteria, published between 2014 and 2025, and printed in English. This timeframe was selected to capture recent trends and developments in sign language and literacy research. Preliminary searches supported this boundary: for example, in SCOPUS, 47 relevant studies were published from 2014 to 2025, compared to 30 between 1960 and 2013, a 53 year timespan. A similar pattern emerged in PsycINFO, with 69 studies published between 2014 and 2025 and 107 during the 53 years prior. These patterns suggest a growing and evolving research base in the last decade, warranting focused attention on more recent interventions and educational strategies. Both qualitative and quantitative studies were included. Additionally, to align with the goals of the current study, articles needed to include an intervention or education strategy and report data regarding its efficacy or effectiveness. Papers were excluded if they did not present and analyze data or otherwise violated the inclusion criteria listed above.

Identification of potentially relevant articles was completed via searching the following bibliographic databases: Academic Search Complete, ERIC, PsycInfo, and Scopus. These four databases were selected because of their complementary and diverse approaches to indexing journals and individual articles. Academic Search Complete, ERIC, and PsycInfo primarily index journals related to education and linguistics. In contrast, Scopus demonstrates a border indexing pattern, which can capture journals or related articles not found in the education-curated databases. The search strategies were drafted by the first two authors. Final search results were exported into a virtual spreadsheet with shared access between all authors, and duplicates were removed.

The search strategy included querying a set of keywords in the full text of peer-reviewed articles in each database. Date and language parameters (i.e., articles published in English between 2014 and 2025) were set before keywords were entered. Keywords were generated by the first two authors using an iterative process that included suggestions and feedback from the third and fourth authors. Keywords included the following sets of terms: “American Sign Language” or “ASL” or “sign language”; “literacy learning” or “literacy development” or “reading development” or “writing development”. Authors individually reviewed each database and extracted data from the identified articles using a virtual spreadsheet form created by the first and second authors. Data on study characteristics (e.g., literacy domain addressed, methodology, interventions, and findings) were abstracted from each article. Data regarding which database search the article appeared in and whether the article contained any duplicates across databases were also included in the shared spreadsheet. Decisions were then made on whether each article met the inclusion criteria.

**Figure 1:**
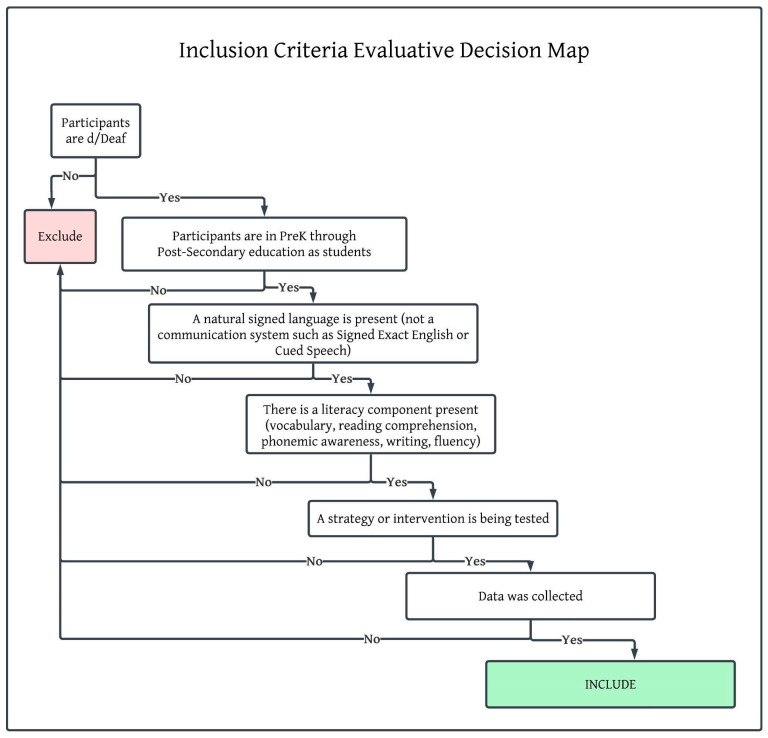
Evaluative decision map for including articles.

The inclusion and exclusion of articles were evaluated using a stepwise process illustrated in Figure 1. Following the initial screening, in which duplicates were identified and removed (n = 52), then the following criteria were applied: First, articles were examined to determine if they contained deaf students as participants, and those that included hearing participants or adults not enrolled in pre-kindergarten through post-secondary education programs were excluded (n = 18). Then, the articles were screened for the use of a sign language (e.g., American Sign Language, New Zealand Sign Language, Indian Sign Language). Articles that included the use of ‘sign support’ ‘visual supports’ or ‘visual communication’ without either naming the language or clarifying the inclusion of the unique grammar, syntax, and features of a natural signed language, as well as those that included the use of a coding system or communication systems, such as Signed Exact English or Cued Speech in isolation, were then excluded (n = 2). Next, articles were screened for relevance by identifying the literacy component present (i.e., any component of writing or reading). No articles were excluded by this criterion, suggesting that our search terms were accurate for narrowing the scope of the presented articles.

**Figure 2:**
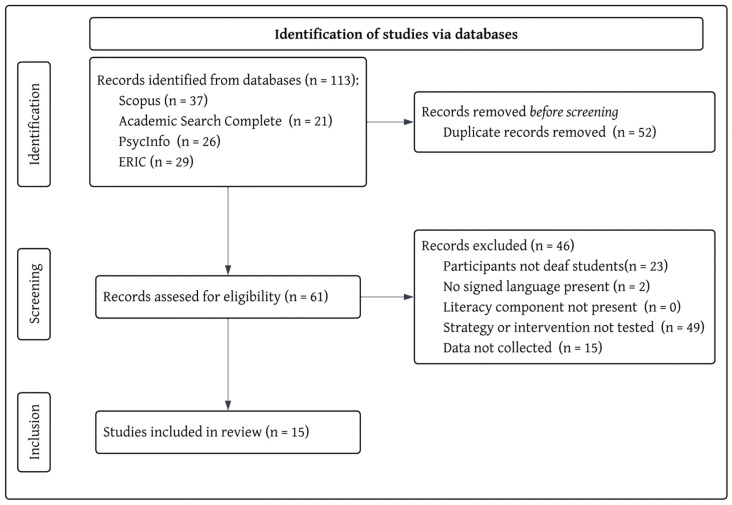
Article inclusion flowchart.

Then, the articles were analyzed to see whether a strategy or intervention had been identified. Articles were included if they identified one or more interventions, curricula, program names, software used, or strategies implemented. In some cases (n = 3 articles), collaborative discussion was initiated to determine if a given article should be included, based on the goals and scope of the current study (Wang & Andrews, 2017; Wang & Andrews, 2021; Ormel et al., 2022). For example, the authors decided that if the participants were attending a bilingual ASL-English education program, those articles would be included as such settings typically reflect a purposeful approach to literacy instruction, where the bilingual education philosophy itself served as the intervention or instructional strategy. Articles that did not mention a strategy, intervention, or use of a bilingual ASL-English model were excluded (n = 49). The last criterion screened was to ensure the article reported and analyzed data. Articles that did not report data on their suggested or trialed interventions were then excluded (n = 15). Following this process, we were left with a total of 15 articles that were appropriate for inclusion in this study. Many articles that were excluded satisfied several exclusion criteria and are reflected in Figure 2.

### 2.2. Analytic Approach

When approaching our analysis of these articles, we used a stepwise process for extracting and working with data. First, we identified overall characteristics across articles that were quantifiable (e.g., the number and demographics of participants and the signed languages used in the interventions or strategies). Next, we divided the corpus in a number of ways: (1) by whether they used quantitative or qualitative research methods, (2) by the type of strategy or intervention they used, and (3) by the overall literacy area they explored (e.g., a component skill such as phonological awareness or vocabulary development, versus a generalized holistic measure of reading, versus composition or writing). By positioning the data in variable ways, our hope was to explore the findings from multiple perspectives. In the section that follows, we present the findings from across all the articles included in this scoping review.

## 3. Results

This scoping review explores the articles that emerged across four databases from 2014 to 2025 regarding the efficacy of strategies and interventions using a signed language that supported literacy learning and development in deaf students in pre-kindergarten through college.

### 3.1. Demographic Characteristics

#### 3.1.1. Participants

The studies included in this review included a total of 415 participants. The study with the smallest number of participants was a single-case design study with one participant (Scott & Hansen, 2020). Conversely, the study with the largest number of participants was 91 (Kuntze, 2023). The average number of participants across all studies was 27.7 students, with the average for the qualitative studies being 15 students and the average number of participants for the quantitative studies being 38.8 students. All studies (n = 15) included at least some students that were in grades K-12, while one study also included postsecondary students (Papen & Gillen, 2024). Articles differed with regard to how they reported the educational status of their students. Six articles reported age alone (Golos & Moses, 2015; Holcomb et al., 2024; Holmer et al., 2017; Ormel et al., 2022; Papen & Gillen, 2024; Tang et al., 2023), six articles reported grade or grade estimate (such as “school age” or “grades K-3” alone (Bajarh, 2020; Dostal et al., 2019; Kuntze, 2023; Wang & Andrews, 2017; Wang & Andrews, 2021; Wolbers et al., 2024)), and three reported age and grade (Falk et al., 2020; Rudner et al., 2015; Scott & Hansen, 2020).

#### 3.1.2. Sign Languages Used

The articles that met our inclusion criteria used a variety of sign languages. The quantitative articles reported the use of the following: ASL (Dostal et al., 2019; Falk et al., 2020; Golos & Moses, 2015; Holcomb et al., 2024; Kuntze, 2023; Scott & Hansen, 2020; Wolbers et al., 2024), Indian Sign Language (Bajarh, 2020; Papen & Gillen, 2024), Chinese Sign Language (Wang & Andrews, 2017; Wang & Andrews, 2021), Swedish Sign Language (Holmer et al., 2017; Rudner et al., 2015), Mexican Sign Language (Kuntze, 2023), Russian Sign Language (Kuntze, 2023), Sign Language of the Netherlands (Ormel et al., 2022), and Hong Kong Sign Language (Tang et al., 2023).

Additionally, one article mentioned the home languages of participants, and although progress and outcomes were measured using American Sign Language and written English, the families of participants were reported to use the following languages: ASL, English, Spanish, Urdu, Tagalog, Portuguese, Bengali (Wolbers et al., 2024). Additionally, one article mentioned the possible presence of simultaneous communication in the educational program of participants, while the primary language of instruction was ASL (Falk et al., 2020). Finally, Wang and Andrews (2017, 2021) also mentioned work in classrooms that used Signed Chinese in addition to Chinese Sign Language. Signed Chinese is a communication system used for pedagogical purposes that places Chinese Sign Language lexical signs in a linear sequence that follows the syntax of spoken/written Chinese. These articles also mentioned the use of Pinyin Fingerspelling and the Chinese Manual Alphabet as separate and distinct components from the signed language used in the studies.

Although literacy in a signed language is important and possible (as outlined in the literature review), students learning to read in their country’s dominant written language are learning to read often in a language they do not use in its spoken format, and the language they do use (their signed language) is not written. Thus, in this review, we found the following written languages as a focus of literacy development: English (Bajarh, 2020; Falk et al., 2020; Dostal et al., 2019; Golos & Moses, 2015; Holcomb et al., 2024; Kuntze, 2023; Papen & Gillen, 2024; Scott & Hansen, 2020; Wolbers et al., 2024), Chinese/Mandarin (Tang et al., 2023; Wang & Andrews, 2017; Wang & Andrews, 2021), Pinyin (Wang & Andrews, 2017; Wang & Andrews, 2021), Swedish (Holmer et al., 2017; Rudner et al., 2015), and Dutch (Ormel et al., 2022). The following articles also mentioned the additional use of the regionally dominant print language in addition to written English (Bajarh, 2020).

Seven articles included participants from bilingual education programs emphasizing a signed and a written language (Dostal et al., 2019; Falk et al., 2020; Holcomb et al., 2024; Kuntze, 2023; Ormel et al., 2022; Tang et al., 2023; Wolbers et al., 2024). One article reported that students received sign language interventions via direct instruction outside of educational environments (Bajarh, 2020), and two articles noted that sign language was used in educational settings via an interpreter or related service provider (such as itinerant teacher of the deaf or speech language pathologist) in a mainstream education setting (Papen & Gillen, 2024; Scott & Hansen, 2020), and two articles reported using sign language within a multimodal framework (Wang & Andrews, 2017, 2021).

### 3.2. Methodological Approach

Among the 15 reviews that met our inclusion criteria, eight of the articles reported using quantitative research methods (see Table 1), while seven reported using qualitative research methods (see Table 2). For the articles reporting on qualitative methodology, all of the articles included unique author teams differentiated by at least one individual, but overlapping authorship did occur in four of the articles (Dostal et al., 2019; Holmer et al., 2017; Rudner et al., 2015; Wolbers et al., 2024). For the articles reporting a quantitative methodology, all of the articles included unique author teams differentiated by at least one individual, but overlapping authorship occurred in four of the articles (Holmer et al., 2017; Rudner et al., 2015; Wolbers et al., 2024). Three articles were identified as “mixed methods” (Bajarh, 2020; Golos & Moses, 2015; Holcomb et al., 2024). We included these in the qualitative results.

In the following section, we will analyze the specific approaches used by each article. However, it is important to note that no single unique combination of methodologies was used twice. The heterogeneity in research goals and participant populations demonstrated a wide variety of approaches each author team adopted. All of the studies, by nature of being included in this scoping review, aimed to explore or support literacy skills in deaf populations. Specific approaches are discussed below.

#### 3.2.1. Quantitative Approaches

First, we examined the approaches used for data analysis that were quantitative in nature. The most overlap in methodology across the quantitative articles was with ANOVA techniques (Rudner et al., 2015; Tang et al., 2023; Wolbers et al., 2024) and *t*-tests (Falk et al., 2020; Kuntze, 2023; Ormel et al., 2022), which appeared across multiple studies (n = 3 for each methodology, respectively) as standard statistical approaches for comparing group differences and measuring changes over time.

ANOVA methods (one-way, multiple factor, MANOVA, etc.) were used by several researchers to examine the relationship between literacy scores and sign language proficiency (Rudner et al., 2015), sometimes with the additional factors of grade level and hearing status (Tang et al., 2023) or effects of an intervention (Wolbers et al., 2024). Similarly, *t*-testing was used by several to test for between-group differences between deaf children with and without cochlear implants (Ormel et al., 2022), between deaf children and hearing children (Tang et al., 2023), between deaf children with hearing or deaf parents (Kuntze, 2023), and between deaf children at different ages (Falk et al., 2020).

Three studies utilized a single case design approach (Holmer et al., 2017; Scott & Hansen, 2020; Tang et al., 2023). Holmer et al. (2017) used a longitudinal cross-over design with measurements at 0, 5, 10, 16, and 39 weeks, and analyzed outcomes using hierarchical linear modeling. Scott and Hansen (2020) used a single case design with a multiple baseline design across measured behaviors. Finally, Tang et al. (2023) used a multiple baselines longitudinal analysis of test performance design and post hoc Tukey tests to determine specific differences between grade levels.

Three of the quantitative studies also used correlation analysis and regression analysis (Kuntze, 2023; Ormel et al., 2022; Wolbers et al., 2024). Ormel et al. (2022) used correlation coefficients to determine predictor variables for word and text reading fluency alongside regression analysis to account for variance of each measured variable at each point of measurement. Wolbers et al. (2024) used Pearson correlation coefficients to measure correlations between expressive language and writing outcomes. Kuntze (2023) used a regression analysis approach to determine the relationship between English reading comprehension and other factors, such as parental hearing status or early language experience.

#### 3.2.2. Qualitative Approaches

Now, we will highlight key similarities across methodologies for the seven qualitative studies that met our inclusion criteria. The majority of the qualitative studies (n = 6) used classroom observations and video recordings to capture classroom interactions (Dostal et al., 2019; Golos & Moses, 2015; Holcomb et al., 2024; Papen & Gillen, 2024; Wang & Andrews, 2017, 2021). Data sources were unique in modality (i.e., visual, textual, and observational), and several studies (n = 3) used triangulation of data through multiple sources such as teacher reports, classroom artifacts, and researcher observations (Golos & Moses, 2015; Holcomb et al., 2024; Papen & Gillen, 2024). One study provided detailed narrative descriptions for teacher interactions with students that presented with variable language profiles (Dostal et al., 2019). The primary focus of these articles described above included the creation of contextual understanding of the classroom environments in which data were collected.

Five studies used narrative qualitative methods. This included the qualitative description of students’ written productions, teacher-student interactions, researcher-student interactions, peer-to-peer interactions, and description of communication modality (Bajarh, 2020; Papen & Gillen, 2024; Wang & Andrews, 2017; Wang & Andrews, 2021). Specifically, Bajarh (2020) used narrative analysis and a pre–post test design to follow the changes of students’ written productions following an interactive writing intervention. Papen and Gillen (2024) employed narrative descriptions to analyze elements that were successful during a peer-guided multiliteracies approach for students. Wang and Andrews (2017, 2021) used qualitative narrative description to detail and reflect upon teachers’ instructional behaviors during literacy tasks with deaf children in China. Finally, one study used grounded theory and narrative methods to analyze the success of an intervention on the production of literacy-related behaviors in young children (Golos & Moses, 2015).

Two studies used discourse analysis as a primary methodology (Dostal et al., 2019; Holcomb et al., 2024). Dostal et al. (2019) used discourse analysis to examine teacher-student interactions in classrooms engaged in writing instruction and learning, and the nature of a specific language strategy that supported successful co-creation of text with deaf students. Holcomb et al.’s (2024) examined classroom discourse as a part of a larger mixed methods approach, using classes as case designs to analyze teacher utterance types, classroom interactions, and the language modalities and proficiencies in the classroom. Aspects of grounded theory were utilized in this article to further propose research-based communication strategies with deaf emergent writers.

### 3.3. Strategies or Interventions Used

Of the fifteen articles that met our inclusion criteria, three articles examined the effects of ASL exposure in a bilingual sign and written language program as the strategy or intervention used (Kuntze, 2023; Tang et al., 2023; Ormel et al., 2022). One article presented the use of bilingual teaching strategies alongside writing instruction to support meaningful connections to English print for deaf college-aged students in India (Papen & Gillen, 2024). Another article examined the use of educational resources in a signed language within a bilingual signed and written language program (Golos & Moses, 2015).

Four articles examined the use of multimodal approaches to literacy that specifically and purposefully utilized sign language (Falk et al., 2020; Scott & Hansen, 2020; Wang & Andrews, 2017, 2021). Scott and Hansen (2020) studied the use of targeted dialogic reading strategies (e.g., content-related thoughtful prompts, targeted questions, meaningful repetition and expansion of a student’s productions to support their literacy acquisition) during instruction in ASL. Wang and Andrews (2017, 2021) examined the use of a signed language and spoken language, as well as speechreading, gestural cues, and Visual Phonics to support literacy skills in elementary-aged students. Finally, Falk et al. (2020) explored the impact of the sight word intervention from the Bedrock Literacy curriculum delivered in ASL (Di Perri, 2013) on silent word reading fluency and vocabulary.

Finally, eight articles mentioned the use of a named curriculum, intervention framework, or specific materials (Bajarh, 2020; Dostal et al., 2019; Falk et al., 2020; Golos & Moses, 2015; Holcomb et al., 2024; Holmer et al., 2017; Rudner et al., 2015; Wolbers et al., 2024). Four of the articles examined writing instruction or composition. Three articles described the use of Strategic and Interactive Writing Intervention (SIWI), an instructional framework that engages students in authentic and meaningful writing opportunities through collaborative, interactive processes with a focus of supporting language and writing, as an intervention (Dostal et al., 2019; Holcomb et al., 2024; Wolbers et al., 2024). Bajarh (2020) described an approach of using holistically meaningful writing activities that supported students’ retention of both content and use in composition through an approach titled “Interactive Writing”.

In addition to the four articles that implemented interactive writing approaches to promote writing skill development, two articles studied the use of computerized software for literacy development (Holmer et al., 2017; Rudner et al., 2015). Both included participants from bilingual schools for the deaf. One article studied software called the “Omega-is-d” software, a version of a previously piloted literacy program for hearing students, was trialed with deaf students (Rudner et al., 2015) through the pairing of Swedish Sign Language and written Swedish. The other article (Holmer et al., 2017) explored the use of computerized sign language-based literacy training, and results indicated an improvement in overall reading skills over time, but the empirical support for the use of the computerized intervention itself was weak (Holmer et al., 2017). Finally, the last article analyzed the implementation of the sight word component of Bedrock Literacy curriculum with students using ASL as their primary language of instruction (Falk et al., 2020).

### 3.4. Summary of Articles’ Results

#### 3.4.1. Component Literacy Skills

In this section, we have subdivided the articles by the component literacy skill they primarily address. Areas of focus aligned with five major components of reading: phonemic awareness, phonological awareness, fluency, vocabulary, and comprehension. Many articles emphasized the importance of multiple literacy skills at once. For these cases, articles were included in the section that most aligned with the authors’ explicit research question. Articles that address written language composition are discussed in a later section. Though an article may focus on a specific component of literacy, each presents findings that have educational implications for language and literacy skills more broadly.

#### 3.4.2. Phonemic Awareness

One qualitative article primarily covered phonemic awareness or the development of a phonetic system for deaf students (Wang & Andrews, 2021). It explored the use of Chinese Pinyin (officially, the Chinese Phonetic Alphabet) as a phonetic system for literacy education in deaf students. It provided an in-depth descriptive analysis of literacy instruction using video recordings in two classrooms, and found that though Pinyin may be a successful tool for educating hearing second-language learners of Chinese, it did not appear to be a successful strategy for deaf students. Instead, visual/tactile approaches were proposed to make phonetic and phonological information accessible for learners. The article suggested that with further research, the use of Pinyin may be accessible to students who maximally benefit from hearing assistive technology, such as cochlear implants. However, at present, Pinyin is not suitable for providing access to auditory literacy materials or instruction for deaf learners.

#### 3.4.3. Phonological Awareness

Two articles, one qualitative and one quantitative, discussed phonological awareness among participants. The first was Holmer et al. (2017), which highlighted children’s ability to imitate unfamiliar lexical signs predicted word reading ability. Authors found that a computerized intervention task used in the research demonstrated weak support for phonological awareness development. Importantly, McQuarrie and Parrila (2014) indicated that signed-language phonology offers a functional and accessible base for literacy acquisition. In sign languages, phonology consists of the same features, timing units, and autosegmental tiers as spoken languages. However, the phonetic units of signed languages consist of contrastive features such as handshape, movement, location, repetition, and palm orientation (Brentari et al., 2018), which function similarly to articulatory features in spoken phonology. Wang and Andrews (2021) found that although teachers use the Chinese phonetic alphabet (Pinyin) as a tool to teach language, both spoken and written Chinese, Pinyin did not serve as a phonological awareness enhancer for deaf students in their classrooms. These findings have implications for phonological awareness as well, given that phonemic awareness and phonological awareness are often connected skills.

#### 3.4.4. Fluency

Two quantitative articles included in this review specifically investigated fluency (Falk et al., 2020; Ormel et al., 2022). In the first study (Falk et al., 2020) authors examined if the sight word component of Bedrock Literacy impacted the fluency outcomes of deaf readers. Findings indicated that participants improved their fluency in reading unfamiliar words following the intervention period. In the second study (Ormel et al., 2022) authors assessed vocabulary knowledge and phonological awareness skills in spoken Dutch and Sign Language of the Netherlands as well as receptive fingerspelling ability, and short-term memory skills. Findings indicated that while spoken language phonological skills contribute to word reading accuracy, they do not significantly influence reading fluency, suggesting that sign-based phonological awareness and short-term memory may play a more prominent role in fluency development, particularly as reading skills develop over time. Additionally, this study found that strong skills in sign language, including the ability to imitate unfamiliar lexical signs and fingerspelling, are positively correlated with word reading and fluency.

#### 3.4.5. Vocabulary

There was one qualitative article that focused on vocabulary (Golos & Moses, 2015) by investigating a children’s media program in ASL. Findings suggested that teacher mediation of video viewing of educational content in ASL paired with the use of follow up classroom activities supported an increase in student’s vocabulary scores, and that curated activities related to ASL content can produce positive results in the appropriate domain (e.g., vocabulary activities produced improvement in vocabulary skills). One qualitative study (Papen & Gillen, 2024) highlighted the importance of developing the depth and breadth of students’ semiotic repertoires via interactive writing approaches to improve their expressive vocabulary use and receptive comprehension efficacy, though vocabulary was not the primary focus of their study.

#### 3.4.6. Comprehension

Of the eight quantitative studies included in this review, four focused on reading comprehension (Falk et al., 2020; Kuntze, 2023; Rudner et al., 2015; Scott & Hansen, 2020). Findings indicated that developing higher-order thinking skills through ASL enhances reading comprehension in deaf students by promoting reasoning and inference-making (Kuntze, 2023). Interventions like Omega-is-d (Rudner et al., 2015) and Dialogic Reading (Scott & Hansen, 2020), which integrate sign language and emphasize multimedia, interaction, and text structure awareness, have shown promise in improving reading skills, including comprehension and sight word fluency. Falk et al. (2020) presented empirical support for the use of the sight word intervention component of Bedrock Literacy for supporting deaf students’ recall of sight words and reading fluency in early and middle grades.

There were two qualitative articles about reading comprehension. One article was specific to reading (Papen & Gillen, 2024), while the other one included both composition and reading (Wang & Andrews, 2017). Papen and Gillen (2024) examined the engagement in interactive, peer-led literacy activities that honored students’ agency and output using authentic texts that were situated in everyday activities. Authors found that use of everyday text relevant to their lives supported students’ development of semiotic repertoires that they were able to functionally use to improve their understanding of other sources they encountered in later lessons. Wang and Andrews (2017) found in the classrooms they observed that academic approaches demonstrated a central focus around speech/hearing and that the curriculum used had lower academic expectations for literacy learning for deaf students than for their hearing peers.

#### 3.4.7. Composition

Among the articles included, there were two quantitative studies focused on writing (Tang et al., 2023; Wolbers et al., 2024), and four qualitative studies focused on writing, three of which were specific to writing (Bajarh, 2020; Dostal et al., 2019; Holcomb et al., 2024) and one that included both reading and writing (Wang & Andrews, 2017). Instructional frameworks like SIWI with a commitment to developing language and literacy simultaneously can support English and ASL development (Wolbers et al., 2024; see siwi.utk.edu). In Holcomb et al.’s (2024) study, teachers using ASL adopted more student-centered, engaging, and responsive strategies, whereas teachers using spoken English tended to rely on whole-class instruction with less individualized support.

## 4. Discussion

### 4.1. The Role of Sign Language in Literacy Acquisition

Collectively, the findings from the studies included in this scoping review emphasize the importance of early access to fluent sign language models, the integration of sign language in literacy instruction, and the need for more targeted research in multilingual co-enrollment settings where signed, written, and spoken languages are part of literacy learning.

Articles that met our inclusion criteria highlight the critical role of language access and instructional alignment in the development of reading and writing skills among deaf students. Despite the implementation of effective teaching methods, the continued reliance on spoken language, particularly in classrooms where simultaneous communication or nonlinguistic communication systems such as Cued Speech or manual spelling alone are used, often creates barriers that hinder deaf students’ full engagement and equitable participation (Marmor & Petitto, 1979; Tevenal & Villanueva, 2009). These obstacles underscore the necessity of providing contextually relevant, structured, and recursive reading and writing support that reflects the linguistic and cultural experiences of deaf learners (Simms & Thumann, 2007).

Articles in this review underscore the foundational role of sign language in supporting component literacy skills, including phonological awareness, vocabulary, and fluency. Quantitative studies show that specific sign language knowledge, such as fingerspelling and the imitation of unfamiliar lexical signs, are positively associated with word reading and reading fluency (Holmer et al., 2017; Ormel et al., 2022). In bilingual educational contexts where both sign and spoken languages are used, English vocabulary and fingerspelling predict reading fluency, while speech phonology contributes more to word-level accuracy than to fluency. These findings suggest that sign-based phonological awareness and short-term memory may become more influential as literacy skills mature. Studies further reveal that sign language phonology provides a rich functional foundation for literacy acquisition (Keck & Wolgemuth, 2020), especially for children who may not have full access to spoken phonology (McQuarrie & Parrila, 2014).

Based on the articles included in this review, there is support for the critical role of accessible early language exposure, particularly a natural signed language, in fostering the literacy development of deaf students. Additional benefits mentioned in these studies included cognitive, social, and linguistic benefits that supported students in their holistic academic endeavors. Early sign language exposure enables children to engage in complex thinking and reading tasks, as it supports the development of metacognitive skills. These skills later support students’ ability to reason, acquire vocabulary, and make meaning from text (e.g., Golos & Moses, 2015). More specifically, instructional approaches that incorporate sign language show promising results for improving reading comprehension and fluency among deaf students. Dialogic reading, an approach that encourages interactive discussions about text, proves especially effective in supporting deaf students as they explore text structures and recall information from text (Scott & Hansen, 2020). These studies collectively affirm other research (e.g., Andrews & Baker, 2019; Bailes, 2001) that integrating sign language into instruction is not only beneficial but essential for promoting literacy among deaf learners.

The use of a signed language not only supports reading development but also offers meaningful pathways for vocabulary and print recognition through educational media and classroom activities. For example, the integration of ASL with educational videos and corresponding classroom materials has been shown to help children connect ASL with English print, thereby enhancing early literacy outcomes (Golos & Moses, 2015). In contexts like China (where several written systems are present in text), tools such as Pinyin are used differently for deaf learners than for hearing peers; while Pinyin supports language learning, it may not enhance phonological awareness in the same way for deaf students, especially those who rely more heavily on visual and tactile modes of learning (Wang & Andrews, 2021). These findings highlight the importance of early and rich exposure to fluent sign language models and the integration of sign language in literacy instruction to support the diverse needs of deaf learners across global educational contexts.

### 4.2. The Case for Sign Language Oriented Classrooms

The majority of deaf students are educated in mainstream classrooms with a general education teacher as their primary teacher (Gallaudet Research Institute, 2012). While these students may have access to sign language, perhaps via an interpreter, itinerant deaf education teacher, or another educational specialist (Papen & Gillen, 2024; Scott & Hansen, 2020), this is not equivalent to receiving direct academic instruction from a sign-fluent model (Kurz et al., 2015; Scott et al., 2021). For many students in these classrooms, sign language may be thought of as an added tool for access, rather than as a language that should be planned for (Nover, 2000).

This is in opposition to the experiences of deaf students in bilingual/bimodal education programs. In these spaces, when at their ideal, sign language holds equal merit with written language, with both languages systematically taught as part of the curriculum by teachers fluent in both modalities, creating an environment where the sign language is recognized as a complete linguistic system with its own grammar, literature, and cultural context (Clark et al., 2020; Humphries, 2013; Scott et al., 2021). As shown above, the incorporation of sign language into the educational lives of deaf children appears to improve many facets of literacy learning, such as phonemic awareness (Wang & Andrews, 2021), phonological awareness (Holmer et al., 2017; McQuarrie & Parrila, 2014), fluency (Ormel et al., 2022), vocabulary (Golos & Moses, 2015), comprehension (Falk et al., 2020; Kuntze, 2023; Rudner et al., 2015; Scott & Hansen, 2020), and composition (Bajarh, 2020; Dostal et al., 2019; Holcomb et al., 2024; Tang et al., 2023; Wolbers et al., 2024). Though our findings here demonstrate a need for replication studies, especially for those elements of literacy that have only one or two articles demonstrating a link between sign language and the assessed area, it is also important to note the overall volume of studies that demonstrate that sign language benefits deaf learners as they develop literacy skills in the dominant written language of their community.

## 5. Conclusions

The reviewed literature highlights a growing body of research using diverse methodological approaches to investigate literacy development and instructional strategies for deaf learners. Quantitative studies predominantly use statistical techniques such as ANOVA, *t*-tests, correlation, and regression analyses, often within longitudinal or multiple-baseline designs, to examine group differences, intervention effects, and predictors of literacy outcomes. These approaches have allowed researchers to identify relationships between language proficiency, particularly sign language exposure, and various component skills within literacy. Notably, multilingual/multimodal educational programs emerge as promising frameworks supporting holistic literacy development by embedding accessible and meaningful language experiences.

Qualitative methodologies complement these findings by providing contextual insights into classroom interactions and student experiences. Narrative descriptions and discourse analysis show how instructional strategies such as interactive writing, dialogic reading, and peer-guided multiliteracies foster holistic and ecologically valid literacy acquisition in classroom contexts. These qualitative approaches emphasize the importance of multimodal communication and teacher–student dynamics, demonstrating how tailored, accessible language interventions promote deeper engagement and literacy growth.

Across both quantitative and qualitative studies, early and sustained exposure to sign language consistently surfaces as a critical factor influencing literacy success. Literacy interventions that purposefully attend to language offer promising avenues for supporting deaf readers and writers, yet the evidence also underscores the complexity of literacy development and the need for continued innovation in assessment and instruction.

Together, these findings advocate for integrated multilingual literacy instructional frameworks that respect the linguistic diversity of deaf students. Future research should continue to build on these foundations by exploring literacy interventions and expanding longitudinal investigations that capture the dynamic interplay between language access, language exposure, language skill development, underlying cognitive processes, presence and degree of language deprivation, and academic achievement.

## Figures and Tables

**Table 1:** Qualifying quantitative articles.

Citation	Country of Participants	Literacy Area of Focus	Research Area/Goals/Aims	Number of Participants	Age of Participants	Participant Hearing Levels	Participant Languages Used	Analytic Approach	Results	Educational Implications
Holmer et al. (2017)	Sweden	Reading	Whether computerized sign language-based literacy training improves reading skill in deaf signing children.	16 (8 boys, 8 girls)	~10.1 years	Not reported (participants attended educational program for the deaf)	Swedish Sign Language, Swedish	Hierarchical linear modeling, longitudinal cross-over design	Ability to imitate unfamiliar lexical signs predicted word reading; intervention showed weak support; suggests supramodal mechanism in word reading development	Reading skills, in particular word reading, are linked to sign language skills.
Kuntze (2023)	USA	Reading Comprehension	Whether ASL comprehension skills, English vocabulary paired with ASL inference skills, or age at enrollment at a bilingual school, home language, and/or quality of the home communication system predict ASL and English comprehension abilities.	91	Middle school aged	Not reported (participants attended educational program for the deaf)	ASL, Mexican Sign Language, Russian Sign Language, English	Multivariate analyses of variance, *t*-tests	Inferencing skills in ASL predict reading comprehension ability. Communication access at home adversely impacted reading skill.	Development of higher order thinking skills in ASL promotes reading comprehension. There is positive impact for instruction of higher order skills in ASL to promote engagement with text and literacy outcomes.
Ormel et al. (2022)	Netherlands	Reading fluency and decoding	How word and text reading accuracy and fluency are impacted by cognitive-linguistic ability in deaf children in bimodal-bilingual education programs.	62	8–10 years	Severe to profound	Sign Language of the Netherlands, Dutch	Longitudinal predictive study with multiple regression analysis.	Fingerspelling ability and speech-based vocabulary strongly predict reading fluency; speech phonology affects word reading accuracy but not text fluency	Children use multimodal (manual and auditory/verbal) skills to address and develop reading skills in bilingual-bimodal educational contexts. More research is needed regarding specific signed and spoken contributors to literacy development.
Rudner et al. (2015)	Sweden	Reading	Whether the pilot, sign language version of the Omega-is literacy software (Omega-is-d1) impact literacy outcomes in classrooms where sign language is the primary mode of communication.	12	~9:6 students in grades 1–2 (n = 6), students in grades 4–6 (n = 6)	Not reported	Swedish Sign Language, Swedish, European and non-European oral and signed languages, home sign systems	Small sample intervention study with pre/post assessment. ANOVA, repeated measures ANOVA.	Substantial literacy skill improvements over 20-day training period. Intervention effect indeterminate from regular classroom activities.	Literacy skills of deaf beginning readers improved substantially throughout this study. Future interventions for deaf beginning readers that utilize sign language as a component should extend the duration of training and fully implement salient practices related to multimedia interaction and recasting.
Scott and Hansen (2020)	USA	Reading comprehension	Whether dialogic reading is a successful intervention for deaf students to improve their comprehension of informational text	1	11 years old, 5th grade	Participant had bilateral cochlear implants, indicating likely past severe hearing levels	American Sign Language, English	Multiple baselines, single case study design	Dialogic reading prompts for recall, identification of text features, and WH comprehension. No interpretable effect of distancing questions due to apparently spontaneous generalization of baseline skill. Evidence of carryover of dialogic reading skills between tiers of intervention despite staggered exposure of skills in intervention.	Dialogic reading may be a successful intervention for supporting comprehension of informational text with deaf students. Especially with the teaching of text structures to improve the accuracy of basic information recall. Author comment mentioned findings intended for students with severe-to-profound hearing levels.
Tang et al. (2023)	China	Writing	Whether deaf children’s exposure to Hong Kong Sign Language in a bilingual co-enrollment environment affect their development of the grammatical features of written Chinese.	29	~7.1 years	Severe-to- profound hearing level	Hong Kong Sign Language, Spoken Cantonese, Written Mandarin Chinese	Multiple baselines longitudinal analysis using two-way ANOVA, one-way ANOVA, *t*-tests, post hoc Tukey tests.	Deaf students initially lagged behind their hearing peers but narrowed the developmental gap by year Primary 2 and caught up in most grammatical features and constructions by Primary 4.	Knowledge of grammar is an essential component supporting literacy skill development. More research is needed regarding whether enrollment in a Sign Language Co-Enrollment environment can support native-like competency in highly complex structures (e.g., relative clauses, location constructions) in written Chinese.
Wolbers et al. (2024)	USA	Writing	The extent to which the use of Strategic and Interactive Writing Instruction (SIWI) improves students’ use of genre-specific traits (across narrative, expository, and persuasive text) in expressive signed and written language. Whether there is a relationship between students’ use of genre-specific traits in expressive signed and written language.	69	Grades 3–6	mild to severe hearing levels	American Sign Language, English. Languages spoken in students’ homes: Spanish, English, Urdu, Mandarin, Tagalog, Portuguese, Bengali	Quasi-experimental study with comparison group. Repeated measures ANOVA, Pearson correlation coefficients	Students in the treatment group made significant gains in expressive and written language for recount and information genres compared to the comparison group, with expressive language and writing positively correlated across all genres at both time points.	Literacy instruction that intentionally integrates expressive language learning with writing supports students’ overall writing and language growth.
Falk et al. (2020)	USA	Reading comprehension and fluency	Whether participation in the Bedrock sight word intervention program impacts the number of sight words or rate at which deaf students are able to read in isolation.	30	6–12 years (grades 1–7)	Variable: moderate hearing levels (n = 1), severe hearing levels (n = 6), profound hearing levels (n = 22), could not test (n = 1)	American Sign Language, English. Author noted occasional Simultaneous Communication may have been included in educational program.	Parametric approach. Pretest/post-test design. Paired sample *t*-tests.	Significant increase in the number of words participants were able to identify from pretest to post-test. Significant increase in raw score performance on Test of Silent Word Reading Fluency (TOSWRF). Significant increases found for grade and age-equivalency scores. Pearson product-moment correlations indicated no significant correlation between TOSWRF performance and the age or grade level. Independent-samples *t* tests confirmed that neither home language nor gender were significant predictors of performance on the TOSWRF.	This study offers empirical evidence to support anecdotal reports of the Bedrock Literacy curriculum’s success with deaf students. Participants in this study demonstrated significant growth in the number of sight words they could read as well as in their fluency in reading familiar words. Unfortunately, the results also offered evidence that Deaf students have difficulty acquiring reading skills.

**Table 2:** Qualifying qualitative articles.

Citation	Country of Participants	Literacy Area of Focus	Research Area/Goals/Aims	Number of Participants	Age of Participants	Participant Hearing Levels	Participant Languages Used	Analytic Approach	Results	Educational Implications
Bajarh (2020)	India	Writing	Whether the Interactive Writing approach—when mediated through Indian Sign Language—is effective in developing the written English skills of deaf students learning English as a second language.	25	9th and 10th grade	Profound hearing loss	Indian Sign Language, written English, print language of dominant societal language for each participant	Narrative inquiry with statistical analysis of writing development.	Statistically significant improvement in writing parameters, better organization and content generation, improved attitude toward writing from students. Challenges with morphological structures, especially inflectional bound morphemes.	Use of sign language with the Interactive Writing approach could be effective with regard to improving both quantity and quality of deaf students’ writing. Specific gains when given contextually relevant, appropriate, structured and recursive writing support.
Dostal et al. (2019)	USA	Writing	Description of an evidence-based approach for engaging and supporting deaf students. Describes a framework that can be utilized to classify student language productions and scaffold them according to their level of clarity and fluency to support the strengthening of languaging.	14 classes of students (exact number of students not reported)	Grades 3–5	Not reported	American Sign Language, English	Discourse analysis and narrative description of classroom observations.	The Language Zone Flowchart provides a framework of support for recognizing and responding to student productions. The strategic use of this approach has the potential to support writing.	The current study presents the Language Zone Flowchart, an instructional tool that can be used to support teachers who aim to engage in developing writing and language skills of students. Use of this framework can support student engagement and strengthen the metalinguistic awareness of students.
Golos and Moses (2015)	USA	Early literacy skills	Whether children’s target vocabulary scores increase after viewing videos and participating in video related activities. Delineating the frequency and type of literacy-related behaviors that children demonstrate during video-related classroom activities. The nature of teacher perceptions about activity efficacy on student literacy learning.	7	3–6 years	Hearing to profoundly deaf	American Sign Language, English	Grounded theory and narrative inquiry via a case study approach with video recording analysis and a teacher survey.	Children displayed targeted literacy skills during activities; descriptive statistics showed higher mean scores in targeted skills after activities	Findings indicate that supplemental media classroom activities can facilitate the learning of literacy skills and help children make connections between ASL and English print. This study offers new opportunities for the integration of early language exposure and curricular materials, and suggests a promising approach to developing key literacy and language skills with research-based media and media-related activities and materials
Holcomb et al. (2024)	USA	Writing	The way in which teachers navigate communication with deaf students who are emergent writers and lack language proficiency during language and writing instruction in grades 3–6, as well as the way in which communication differs between teachers who primarily use spoken English and those who primarily use ASL.	9	8–13 years	Variable; moderately severe (n = 4), severe (n = 1) profound (n = 4) with variable use of hearing assistive technology.	American Sign Language, English	Narrative inquiry, classroom observation, analysis of language modalities and communication patterns.	ASL-using teachers employed more student-centered approaches with greater engagement and communication learning; spoken English-using teachers favored whole-class instruction with less individualized support	Despite the high quality of the teaching methods used in classrooms where teachers primarily used spoken language, students experienced decreased engagement which served as a barrier toward equal opportunities in their learning. Deaf emergent writers in classrooms where sign language support was inconsistent (e.g., settings that used SimCom) demonstrated less engagement as well. Teacher preparation programs and professional development must emphasize the skills necessary to engage all deaf students to ensure educational equity.
Papen and Gillen (2024)	India	Reading	Whether the use of peer-guided engagement with contextually appropriate multiliteracies encountered in the daily life of students can support their development of literacy skills.	39	16–30 years	Not reported. Authors reported none of the participants or peer guides used hearing assistive technology.	Indian Sign Language, written English	Narrative inquiry, case study analysis across several peer-guided small group classes. Discussion of insights from pilot initiative using peer-guided literacy activities.	Students’ semiotic repertoire grew throughout the experience, and this contributed to their later contributions and written production. Inclusive educational approaches that utilize the benefits of peer-guided literacy maintain engagement and support carryover of skills across sessions.	This project shows the value of a deaf-led inclusive education where sign language and literacy are supported and developed, not used exclusively as a means to produce spoken language outcomes. Practitioners and policy-makers should seek to develop education opportunities for deaf people grounded in the value of diversity as a strength and building on deaf children and adults’ agency.
Wang and Andrews (2017)	China	Reading and writing	What methodologies are used to engage deaf students in literacy activities during classroom instruction.	11	Grades 1–2	Not specifically mentioned, but classrooms included deaf students with hearing teachers.	Chinese Sign Language, spoken Chinese, spoken English. Authors also mentioned the use of Signed Chinese, Chinese character signs, Pinyin, and Pinyin Fingerspelling	Narrative inquiry, video analysis of classroom instruction.	Instruction followed the Gradual Release of Responsibility model with a focus on speech and hearing outcomes. The curriculum had lower academic expectations for deaf students than for their grade-matched hearing peers.	The content load of curriculum must be increased for deaf students to raise the academic performance expectations of these students. Deaf education programs would benefit from hiring more deaf teachers to provide appropriate language models. Use of storybooks can be adapted and implemented with deaf children to support background knowledge development.
Wang and Andrews (2021)	China	Reading	The extent and nature of the role Chinese Pinyin plays in deaf education classrooms, specifically during literacy instruction and the nature of its impact on deaf students’ literacy outcomes.	11	Grades 1–2	Not listed. Authors stated students were deaf.	Chinese Sign Language, Chinese manual alphabet, Chinese finger syllabary, spoken and written Chinese.	Narrative inquiry with the use of transcribed discourse from video recordings in the classroom.	Pinyin serves different functions for deaf students versus hearing students. Visual and visuo-tactile approaches make phonological information accessible to deaf students, not necessarily mastery of Pinyin.	For DHH children, Pinyin does not serve as a phonological awareness enhancer, but teachers use it as a tool to teach language, both spoken and written Chinese. Pinyin instruction has been found to benefit hearing Chinese L2 learners. The increased use of hearing assistive technology may support student learning of Pinyin and Pinyin fingerspelling/Chinese manual alphabet as a tool to “bootstrap” the learning of spoken Chinese.

## Data Availability

Not applicable.
